# Validity and Reliability of a New Inertial Device for Monitoring Range of Motion at the Pelvis during Sexual Intercourse

**DOI:** 10.3390/ijerph17082884

**Published:** 2020-04-22

**Authors:** José M. Oliva-Lozano, Isabel Martín-Fuentes, José M. Muyor

**Affiliations:** 1 Health Research Centre, University of Almería, 04120 Almería, Spain; jol908@ual.es; 2 Laboratory of Kinesiology, Biomechanics and Ergonomics (KIBIOMER Lab.), Research Central Services, University of Almería, 04120 Almería, Spain; imf902@ual.es

**Keywords:** sexual activity, posture, IOC, kinematics, WIMU

## Abstract

To understand the physical demands of sexual intercourse, it is necessary to monitor the kinematic parameters of this activity using relatively non-invasive technology. The aims of this study are to analyze the validity and reliability of an inertial device for monitoring the range of motion at the pelvis during simulated intercourse and compare the range of motion (ROM). Twenty-six adults were monitored during intercourse using an inertial device (WIMU) and a motion capture system (gold standard) in a test that consisted of 4 sets of 20 simulated in–out cycles (IOC) in missionary and cowgirl positions. Men and women were tested separately in a laboratory setting for simulated intercourse aims. There were no differences between the WIMU and the gold standard system at fast pace (*p* > 0.05), whereas there were differences at slow pace (~2.04°; *p* ≤ 0.05; *d* = 0.17). Intraclass correlation coefficients (ICCs) for the relationship between systems was very close to 1 at both paces (slow: 0.981; fast: 0.998). The test–retest reliability analysis did not show any difference between sets of measurements. In conclusion, WIMU could be considered as a valid and reliable device for IOC range of motion monitoring during sexual intercourse in missionary and cowgirl positions.

## 1. Introduction

Sexual activity has been recognized as an essential, integral aspect of human life [1]. When practiced safely and well, it offers health benefits and is closely linked to life expectancy [2]. Various studies have shown that sexual activity may have mental health benefits and improve cognitive functioning [3]. It may work as an anti-stress therapy by increasing levels of oxytocin [4], which inhibits the action of cortisol [5]. Sexual activity also decreases the risk of cardiovascular disease [1]. Frequent sexual activity can increase vasodilatory capacity, improve the functioning of the vascular wall of the arteries and veins and improve the efficiency with which oxygen is provided to the muscles, thus promoting cardiovascular health [6].

Sexual intercourse can be considered a physical activity since it involves musculoskeletal movement that results in energy expenditure [7]. Ainsworth et al.’s [8] compendium of physical activities lists sexual activity as having a mean intensity of 1.5 to 1.8 metabolic equivalent of tasks (METs), and a recent study concluded that healthy adults perform sexual intercourse at an intensity of 5.8 METs [9]. However, more research on different kinematic and physiological parameters during sexual intercourse is needed in order to have a better understanding of the demands of this activity [10].

Perhaps part of the reason for the lack of research on sexual intercourse is that it is an activity that involves physical intimacy with another person. In some sectors of the society, decisions about sexual intercourse may carry connotations of acceptance or rejection. The methodological difficulties associated with this type of research may also be a factor. Regardless, the variety of results obtained about the intensity and demands of the sexual intercourse in the studies [7,8,9] carried out to date makes it necessary to re-examine the physical demands of sexual intercourse. The use of less invasive technology, which takes into account the bioethical issues involved in this type of research, could allow genuine (not simulated in the laboratory) sexual intercourse to be monitored.

Several studies have tried to monitor the lumbar spine range of motion during sexual intercourse in different men’s and women’s positions [11,12] since some of the factors related to a decrease in the frequency of sexual intercourse are not only physiological but also mechanical [12]. For example, a previous study reported that the second and third most frequently quoted statements concerning the decrease in the frequency of sexual intercourse by females and males, respectively, was “difficulty with pelvic movements” [11,13]. In this regard, previous investigations on the kinematic demands of sexual intercourse concluded that positions such as the missionary elicited high lumbar spine flexion in both men and women, and therefore, it was not recommended for the flexion-intolerant patient [11,12]. As well as this, another investigation observed that the cowgirl position required intensive hip flexion range of motion, which caused prosthetic impingements [14]. As a result, this intercourse position could be potentially risky for patients with hip pathologies [14]. Hence, a biomechanical analysis of the movements and postures during sexual intercourse is considered necessary, specifically for the mentioned-above positions [11,12,14]. In addition, the ability of a system to calculate the range of motion of the lumbar spine implies its ability to detect the in–out penetration cycles (IOCs), described as the angular displacement from maximum flexion to maximum extension of the pelvic movement [12]. Gold standard motion capture (MOCAP) systems are used in this type of research, but the use of these systems is restricted to laboratory settings for technical reasons: the complexity of the installation, calibration procedures, or data analysis [15].

Therefore, at the moment, it appears that inertial measurement units (IMUs) may be a good alternative to MOCAP systems. IMUs collect 3D data (x, y, and z) using a combination of accelerometers, gyroscopes, and magnetometers [15]. In addition, some IMUs may synchronize with physiological data collected from additional sensors (e.g., heart rate, muscle activation, or muscle oxygen sensors) [16]. This allows practitioners to gain a better understanding of both the physiological and kinematic demands of sexual intercourse. Thus, the use of wearable sensors is essential when doing research in a sexual activity context. These devices are wireless, light, small, and easy to use [15]. Consequently, participants have intimacy and autonomy to perform the activity at home without the intervention of any researcher or specialist. Moreover, the multi-sensor fusion of accelerometers, gyroscopes, or magnetometers may be beneficial to successfully comprehend the participants’ performance in sexual intercourse [17]. Various systems are currently available on the market for research purposes and clinical applications [15], but several improvements in data logging, data processing, and device attachment must be made before these systems can be used more widely [18].

Given the above-mentioned reasons regarding the methodological difficulties associated with this type of research, the importance of measuring range of motion during specific sexual intercourse positions (e.g., missionary and cowgirl positions) as well as the advantages of the use of wearable sensors (i.e., practically useful to monitor real situations without causing an invasion to the participants), the validity and reliability of inertial devices for their use during sexual intercourse is necessary. Only one study has investigated the application of accelerometers to sexual intercourse in order to monitor inertial parameters that may help to accurately classify sexual disorders (e.g., premature or delayed ejaculation) [19]. However, we do not know any studies that have analyzed the validity and reliability of inertial devices when used to evaluate the range of motion during sexual intercourse. Hence, the aims of this study are (1) to analyze the concurrent validity of an inertial device for monitoring range of motion at the pelvis during simulated intercourse in missionary and cowgirl positions; and (2) to analyze the test–retest reliability of an inertial device for monitoring range of motion at the pelvis during simulated intercourse in missionary and cowgirl positions.

## 2. Materials and Methods

### 2.1. Participants

Twenty-six participants (age: 23.65 ± 3.01 years old; height: 1.75 ± 0.07 m; weight: 70.75 ± 12.43 kg) took part in the study, 15 men (age: 24.2 ± 3.02 years old; height: 1.79 ± 0.06 m; weight: 77.86 ± 10.47 kg) and 11 women (age: 22.91 ± 2.94 years old; height: 1.69 ± 0.04 m; weight: 61.04 ± 7.3 kg). The sample size was calculated using G*Power software (Heinrich-Heine-Universität Düsseldorf, Düsseldorf, Germany) [20], specifying statistical power >0.85, *p* < 0.05, and medium effect size (Cohen’s *d* = 0.5) for sufficient power in the study [21]. The aims and methods were explained to all participants, and any questions they had about the procedures or other aspects of the study were answered. They were told that they would be free to stop the test or leave the study at any time. They then provided fully informed consent to take part in the study. Prior to the evaluation, all participants were informed verbally and in writing of the study objectives and procedures. The procedures were previously designed according to the Declaration of Helsinki and approved by the University’s Bioethics Committee.

The inclusion criteria were male or female; adults between the ages of 18 and 55 years old; previous experience of heterosexual intercourse in the positions specified in the Methods section. Any diagnosed pathology or musculoskeletal dysfunction was considered as grounds for exclusion.

### 2.2. Procedure

Potential participants received a dossier giving information about the objectives and protocol of the study so that they would know what was involved before attending the laboratory appointment. Men and women were tested singly in separate sessions. Potential participants who were available and interested in participating were assigned to the appropriate test session. The protocol was explained again, with a demonstration by one of the male researchers (for the men’s session) and by a female collaborator (for the women’s session).

**Figure 1:**
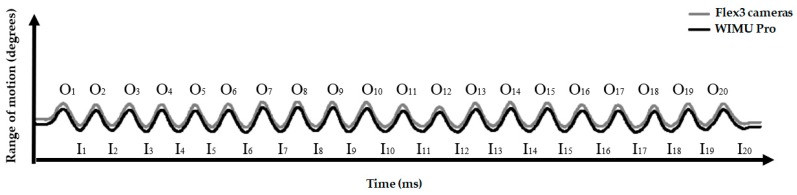
Description of the range of motion from twenty in–out cycles (IOCs) that were performed by one participant recorded by both systems (Flex 3 cameras and WIMU Pro) and separated by in–out phases. (I: in-phase; O: out-phase).

To ensure that participants would be comfortable performing the required movements, folding screens were placed between the researchers and participants to prevent the researchers from seeing the movements made during the tests and thus give participants some privacy. A female collaborator was present in the laboratory throughout the women’s tests, even though the researchers could not see the movements of participants. The participants performed 4 sets of 20 in–out cycles (IOCs; 2 sets at a slow pace and 2 sets at a fast pace) after 1 familiarization set to the test at each pace. Sidorkewicz and McGill [12] describe the IOC as the range of motion (degrees) between the maximum flexion and maximum extension of the movement (Figure 1). Then, the range of motion from each IOC was collected.

**Figure 2:**
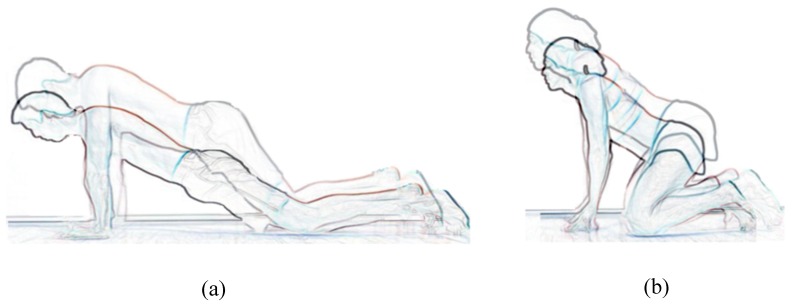
(**a**) Missionary posture. (**b**) Cowgirl posture.

The participants performed the sets back-to-back with a five-second break staying in the “out” phase of the cycle. The absolute frequency of movement at both paces was controlled by the participants in accordance with their previous experience. Thus, the familiarization sets were used to allow participants to adapt themselves to the procedure. The data collected during the familiarization sets were excluded from the statistical analysis. The included sets (Sets 1 and 2 for slow and fast paces) were used to analyze concurrent validity, whereas the test–retest reliability was analyzed by following the same protocol on a different session. Men performed the test in the “missionary” posture (Figure 2a) and women in the “cowgirl” posture (Figure 2b). After the test had been completed, the recorded data were encrypted in a database.

### 2.3. Instruments

One WIMU Pro device (RealTrack Systems, Almería, Spain) was used to record pelvic movements. This device incorporates a variety of inertial sensors, which include four 3D accelerometers and three 3D gyroscopes that collect data at a sampling frequency of 1000 Hz in addition to a 3D magnetometer and a barometer that collects data at 100 Hz. This device provides 3D accelerometry data (x, y, z) and 3D angular velocity (x, y, z), and combining these parameters enables the attitude sensor to calculate the orientation of an object with respect to a reference point. Since the movement of a rigid solid with respect to a fixed point is described by the Euler angles, a set of 3 angular coordinates appears, which provide the orientation of a reference system of mobile orthogonal axes with respect to a fixed one [22]. Thus, angular displacement could be analyzed by the Euler Z channel. The device was placed vertically in an elastic pocket (Aptonia, Lille, France) attached to the sacral area.

**Figure 3:**
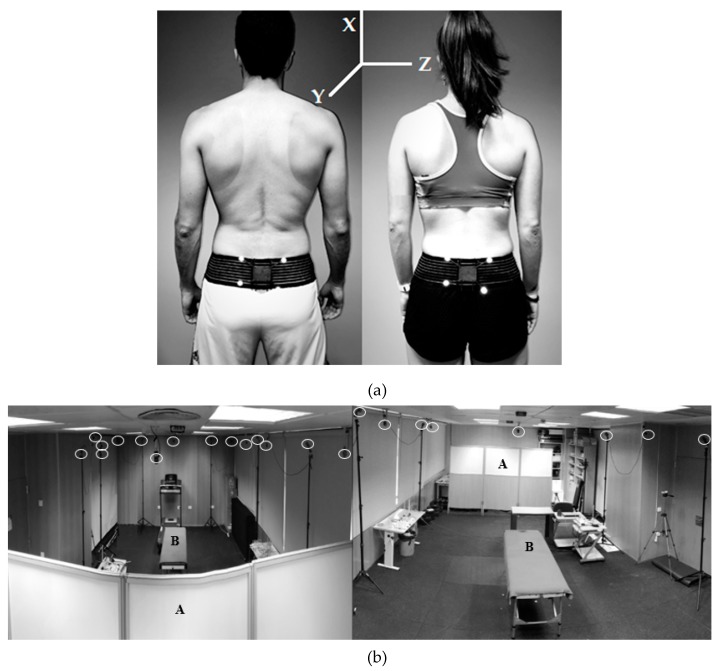
(**a**) Placement of inertial device and spherical markers on the sacrum in men and women. (**b**). Back and front view of the experimental set-up, which included folding screens (A), testing area (B), and 16 optoelectronic cameras (white circles).

A MOCAP system of 16 infrared cameras (Flex 3, Optitrack, Natural Point, OR, USA) was also used (this system is considered as the gold standard) for the first and second aim of the study. This system registered the angular displacement of a rigid body created by 4 spherical markers (B & L Engineering, Tustin, CA, USA) that were placed, by the same tester, on a rigid body marker base which had 4 threaded marker posts of different lengths (Optitrack, Natural Point, OR, USA) on top of the WIMU Pro device in the sacrum area (S3 vertebrae was considered as the anatomical reference for the placement of the rigid body marker base; Figure 3a) within the testing area (Figure 3b). These 4 markers formed a rigid body from which the angular displacement on the *x*-axis was extracted at a frequency of 100 Hz using Motive software (Optitrack, Natural Point, OR, USA). This system has shown good accuracy and reliability in clinical and research applications [23] and spinal morphology testing [24].

In the current study, each IOC was calculated by “Cycles Monitor”, available on SPro software (RealTrack Systems, Almería, Spain). The “Cycles Monitor” analyzes the signal from angular displacement data. It is based on an algorithm that selects windows of samples (*N* = 400) and calculates the midpoint of that window, which is the origin of the signal. Once the origin of the signal allows the identification of both positive and negative phases, the “Cycles Monitor” detects peaks and calculates the difference between one peak and another (range of motion in degrees). Then, the range of motion between the minimum and maximum values (degrees of the in–out phases, respectively) of every IOC was obtained for each instrument

### 2.4. Data Synchronization from Both Instruments

The synchronization of the data from the two systems was made possible by reducing the sampling frequency of the inertial device from 1000 to 100 Hz using SPro analysis software (RealTrack Systems, Almería, Spain). First, the down-sampling procedure was followed with a low-pass filter, which only lets low-frequency data pass through. The software uses the Fast Fourier Transform to analyze the signal in the frequency domain and eliminate the data at high frequencies (more than 100 Hz). Then, the software takes 100 samples from a total of 1000 samples in a second and removes the noise from the signal. Therefore, the synchronization of the data was facilitated since the signals from both instruments were now at 100 Hz. By observation, maximum and minimum peak values of every IOC from both signals were detected. Subsequently, the time offset in milliseconds between one signal and another was calculated. Since both data sources have a constant frequency, SPro software has an “Apply time offset” option that corrects that time difference between both signals. Then, the minimum value (degrees) of the “out” phase of the first IOC of each test and the maximum value (degrees) of the “in” phase of the last IOC in the test were reviewed in order to ensure that the synchronization procedure was successful.

### 2.5. Statistical Analysis

Preliminary Shapiro–Wilk normality tests indicated that all variables (range of motion during IOC at Set 1, 2, 3, and 4 collected by WIMU Pro and Flex3 cameras) were normally distributed, so parametric tests were used in all subsequent analyses.

Student’s *t*-test for paired samples was used to detect systematic differences between the systems (validity), and between the sets performed by a given participant (reliability). Effect sizes for between-groups effects (Cohen’s *d*) were calculated using a combined standard deviation and evaluated using the following criteria, trivial: 0–0.19; small: 0.20–0.49; medium: 0.50–0.79; large: ≥0.8 [25].

The concurrent validity of the WIMU Pro device was analyzed by calculating the following statistics: the difference between the systems (systematic bias), pairwise relationships between the systems (calculated using least squares linear regression [26], standard errors of measurement (SEMs), intraclass correlation coefficients (ICC) (2,1) with 95% CIs [27], and effect sizes (to quantify the magnitude of differences).

ICCs with 95% CI were also used to evaluate the relative reliability of each system in calculating mean angular movement at slow and fast paces. Absolute reliability, defined as the degree to which repeated measurements vary within individuals, was determined using the standard error of measurement (SEM) and the coefficient of variation (expressed as % CV) [28].

The statistical power and effect size were calculated with G * Power software (v.3.1) (Heinrich-Heine-Universität Düsseldorf, Düsseldorf, Germany) for OSX [20]. The statistical power was greater than 0.9 in all the variables analyzed with the sample size selected in the present study. The statistical analysis was carried out with IBM SPSS Statistics software version 25 (SPSS, Inc., Armonk, NY, USA), and the level of significance was set at *p* ≤ 0.05.

## 3. Results

Table 1 shows the concurrent validity of WIMU Pro device as a method of monitoring range of motion in IOCs at slow and fast paces, relative to the gold standard Flex3 camera system. There were differences between the systems at slow paces (~2.04°, *p* < 0.001), but the effect size was very small (*d* = 0.17) and the difference between systems was lower than the standard error of the measurement (SEM = 2.34°). At fast pace, there were no significant differences between the systems (~0.24°, *p* > 0.05) and the differences were lower than the SEM (2.96°). At both paces, the ICC was very close to 1, with *p* < 0.001 (Table 1).

Table 2 shows the test–retest reliability of the systems when used to monitor range of motions during IOCs at slow and fast paces. There was no systematic difference between the test and retest and the absolute differences were lower than 1.5° for both systems. The SEM obtained was 2.6°. The % CV was greater at the slow pace than the fast pace in both systems (mean: 7.72% vs. 5.28%). The ICC was very high in both systems (mean: 0.987, *p* < 0.001).

**Figure 4:**
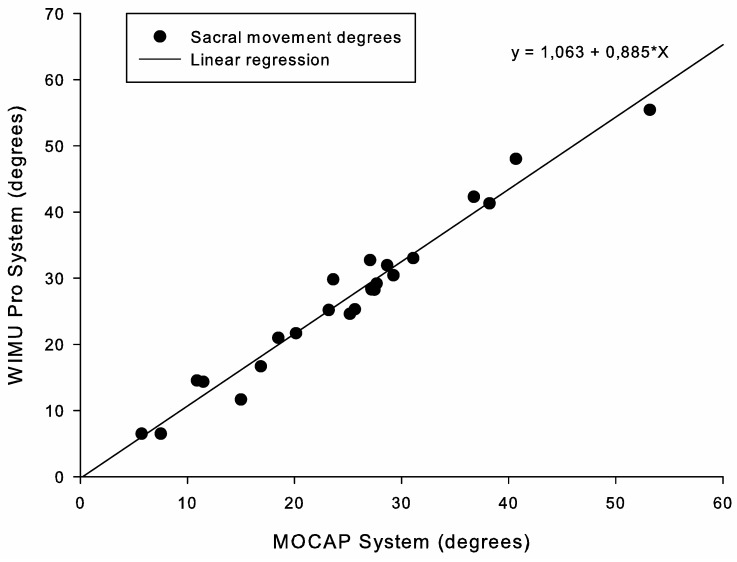
Correlation between systems for range of motion monitoring during slow IOCs.

**Figure 5:**
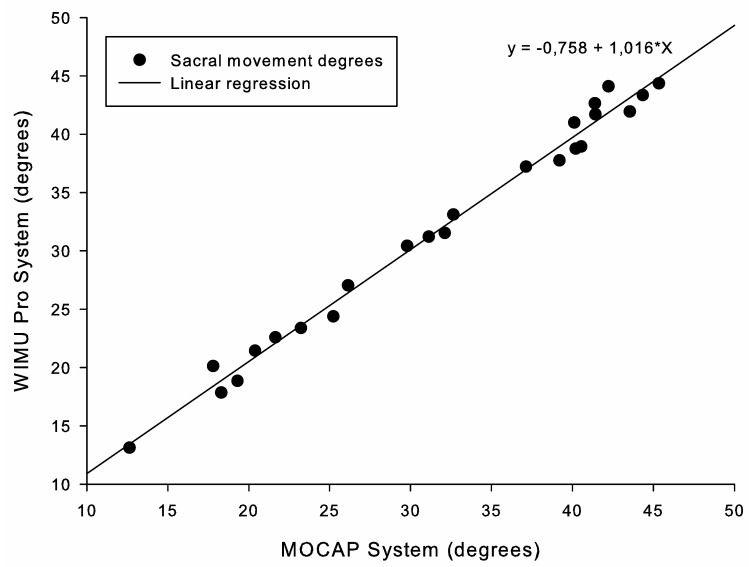
Correlation between systems for range of motion monitoring during fast IOCs.

Figure 4 and Figure 5 represent the results of the linear regression analysis at slow and fast paces, respectively. Both figures show a high positive correlation between the angular values registered by the two systems, R^2^ > 0.9 (*p* < 0.001; Table 1).

## 4. Discussion

The aims of this study are to analyze the concurrent validity and test–retest reliability of an inertial device for monitoring range of motion at the pelvis during simulated intercourse in missionary and cowgirl positions.

The main finding was that the WIMU Pro system produced valid measurements when compared with the gold standard MOCAP system (16 Flex3 infrared cameras). In addition, the WIMU Pro showed high test–retest reliability when used to monitor the range of motion of IOCs in men and women.

The WIMU Pro device can be considered to have high concurrent validity as the difference between the systems was just 2.04°, which was lower than the SEM (2.65°). In addition, the ICC and R^2^ for both paces were greater than 0.96 (*p* < 0.001). A previous analysis of the concurrent validity of WIMU Pro for the analysis of the range of motion during flexion and extension of the hip in a straight leg raise test [29] found similar results (difference = 0.5°, ICC = 0.99, and R^2^ = 0.99), although the SEM was lower (0.05).

Other studies that have validated inertial sensors by comparing them with optical systems for capturing the range of motion in the same axis of movement (flexion–extension) found the following systematic differences in evaluations of the hip movement: 1.55° (during a hip flexion test) [30], 1.8° (during an upright posture test) [31] and 2.42° (during level walking test) [32]; in evaluations of the range of motion of the trunk during a sit-to-walk test: 0.45° [30]; in the range of motion of the lumbar spine during a standing forward flexion test: 1.82° [33], in the range of lumbar–pelvic movement during a standing forward flexion test: 3.06° [21]. The following additional statistics were reported: R^2^ = 0.78 [21] and R^2^ = 0.82 [34]; SEM = 2.47° [32] and SEM = 3° [31,33], and an ICC of 0.99 [35].

It was necessary to assess the test–retest reliability of the WIMU Pro device for monitoring IOCs in men and women to confirm that observed differences in the range of motion were not due to systematic errors of measurement and were not random errors caused by mechanical variation [28]. The systematic bias was less than 0.5° at both paces, and the maximum CV was 7.15% (CVs ≤ 10% are considered acceptable for analytic purposes [28]. The ICCs showed that the WIMU Pro has excellent reliability when used for IOC monitoring. Other researchers who have analyzed the reliability of gold standard technology for analyzing the range of motion of the hip [36] found an ICC of 0.92, CV of 3.6°, and SEM of 1.9°. A previous study of the reliability of the WIMU Pro device when used to measure the range of motion in hip flexion–extension [29] reported an ICC of 0.984, CV of 0.01%, and SEM of 0.31°.

The main limitation of this study is that, because analysis of the validity and reliability of a device requires control over the process of data collection, it had to be carried out under laboratory conditions. For example, the participants simulated the movement individually (not in pairs). Moreover, both IOC paces depended on the participants’ experience in sexual intercourse. The test–retest reliability was analyzed with a five-second break. As this is the first study to analyze the validity and reliability of an inertial device for monitoring range of motion at the pelvis during sexual activity in men and women, the results could only be compared with previous research on the validity or reliability of similar devices testing different variables and a similar range of motion (same axis of movement) [21,29,31,32,33,34,35,36]. Further research on the validity and reliability of other devices for monitoring range of motion at the pelvis during sexual activity is needed to make it easier to compare systems. Furthermore, it would be of great interest to conduct future validation studies for the SPro software algorithms, which are the foundation of any outcome variable. Since this study collected data from young adults (~24 years old), it is suggested that future studies consider a larger sample size, which may include a wide range of ages.

## 5. Conclusions

This research showed that the WIMU Pro is a valid and reliable inertial device for monitoring IOC range of motion during sexual activity in missionary and cowgirl positions. The WIMU Pro could be used to analyze the kinematic parameters of specific forms of sexual activity in naturalistic contexts. However, caution should be taken when analyzing the range of motion at the pelvis in different positions of sexual intercourse.

## Figures and Tables

**Table 1:** Concurrent validity of WIMU Pro device relative to the MOCAP system when used to monitor range of motion during fast- and slow-paced IOCs.

	Slow Pace	Fast Pace
WIMU Pro (95% CI; °)	24.91 ± 10.92 * (19.91–29.27)	31.01 ± 11.96 (24.64–37.83)
Flex3 cameras (95% CI; °)	26.96 ± 12.13 (20.62–29.65)	31.26 ± 11.72 (24.56–37.01)
Systematic bias (°)	−2.04 ± 2.45	−0.25 ± 0.24
Cohen’s *d*	0.17	0.02
SEM (°)	2.34	2.96
R^2^ correlation	0.96 *	0.98 *
ICC (95% CI)	0.981 (0.894–0.994) *	0.998 (0.993–0.999) *

* *p* < 0.001; IOC: in-out cycle; CI: confidence interval; SEM: standard error of measurement; ICC: intraclass correlation coefficient; °: degrees.

**Table 2:** Test–retest reliability of WIMU Pro and MOCAP system when used to monitor the range of motion during fast- and slow-paced IOCs.

Variable	WIMU Pro		Flex3 Cameras	
	Slow Pace	Fast Pace	Slow Pace	Fast Pace
Set 1 (95% CI; °)	24.69 ± 11.31 (19.91–29.27)	31.24 ± 12.37 (24.64–37.83)	26.38 ± 12.57 (21.07–31.69)	31.39 ± 12.13 (24.92–37.85)
Set 2 (95% CI; °)	25.14 ± 10.69 (20.62–29.65)	30.78 ± 11.67 (24.56–37.01)	27.54 ± 11.83 (22.54–32.54)	31.13 ± 11.42 (25.05–37.22)
Systematic bias (°)	−0.44 ± 2.73	0.45 ± 2.50	−1.16 ± 2.72	0.25 ± 2.35
Cohen’s *d*	0.04	0.03	0.09	0.02
SEM (°)	2.24	3.00	2.48	2.94
CV (%)	7.15	5.47	8.29	5.09
ICC (95% CI)	0.985 (0.965–0.993) *	0.986 (0.964–0.994) *	0.989 (0.970–0.996) *	0.990 (0.973–0.997) *

* *p* < 0.001; IOC: in-out cycle; CI: confidence interval; SEM: standard error of the measurement; CV: coefficient of variation; ICC: intraclass correlation coefficient; °: degrees.
